# Genome analysis reveals diverse novel psychrotolerant *Mucilaginibacter* species in Arctic tundra soils

**DOI:** 10.1093/ismeco/ycaf071

**Published:** 2025-04-25

**Authors:** Anil Kumar, Minna K Männistö, Marika Pätsi, Lee J Kerkhof, Max M Häggblom

**Affiliations:** Department of Biochemistry and Microbiology, Rutgers University, New Brunswick, NJ 08901, United States; Department of Marine and Coastal Sciences, Rutgers University, New Brunswick, NJ 08901, United States; Natural Resources Institute Finland, FI-96200 Rovaniemi, Finland; Natural Resources Institute Finland, FI-90570 Oulu, Finland; Department of Marine and Coastal Sciences, Rutgers University, New Brunswick, NJ 08901, United States; Department of Biochemistry and Microbiology, Rutgers University, New Brunswick, NJ 08901, United States

**Keywords:** *Mucilaginibacter*, tundra soil isolates, cold-adapted, novel species

## Abstract

As Arctic soil ecosystems warm due to climate change, enhanced microbial activity is projected to increase the rate of soil organic matter degradation. Delineating the diversity and activity of Arctic tundra microbial communities active in decomposition is thus of keen interest. Here, we describe novel cold-adapted bacteria in the genus *Mucilaginibacter* (*Bacteroidota*) isolated from Artic tundra soils in Finland. These isolates are aerobic chemoorganotrophs and appear well adapted to the low-temperature environment, where they are also exposed to desiccation and a wide regime of annual temperature variation. Initial 16S ribosomal RNA (rRNA)-based phylogenetic analysis suggested that five isolated strains represent new species of the genus *Mucilaginibacter*, confirmed by whole genome-based phylogenomic and average nucleotide identity. Five novel species are described: *Mucilaginibacter geliditolerans* sp. nov., *Mucilaginibacter tundrae* sp. nov., *Mucilaginibacter empetricola* sp. nov., *Mucilaginibacter saanensis* sp. nov., and *Mucilaginibacter cryoferens* sp. nov. Genome and phenotype analysis showed their potential in complex carbon degradation, nitrogen assimilation, polyphenol degradation, and adaptation to their tundra heath habitat. A pangenome analysis of the newly identified species alongside known members of the *Mucilaginibacter* genus sourced from various environments revealed the distinctive characteristics of the tundra strains. These strains possess unique genes related to energy production, nitrogen uptake, adaptation, and the synthesis of secondary metabolites that aid in their growth, potentially accounting for their prevalence in tundra soil. By uncovering novel species and strains within the *Mucilaginibacter*, we enhance our understanding of this genus and elucidate how environmental fluctuations shape the microbial functionality and interactions in Arctic tundra ecosystems.

## Graphical Abstract

**Graphical Abstract:**
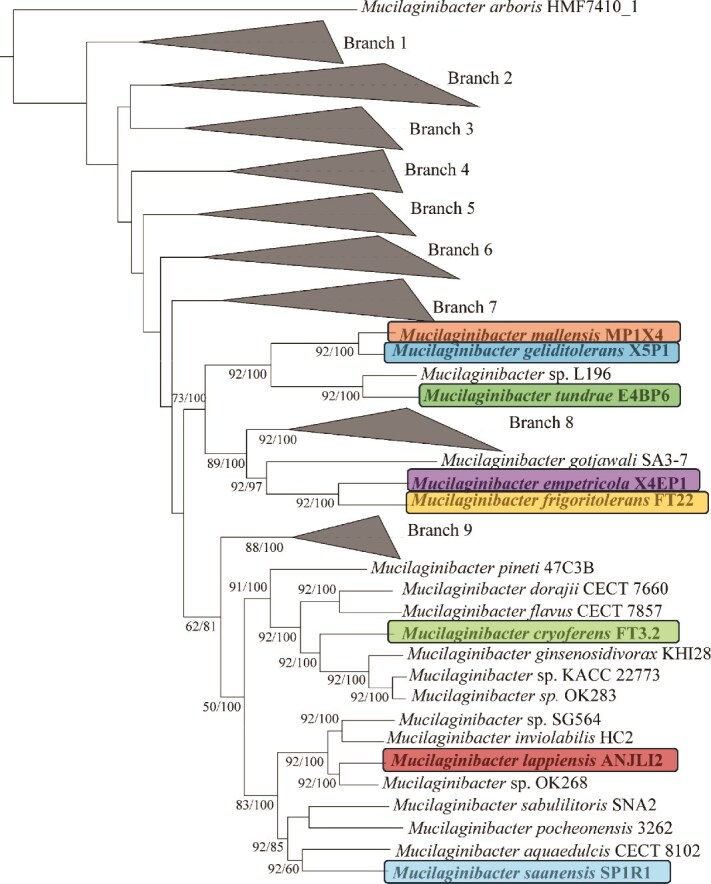

## Introduction

More than one-third of the global organic carbon pool is stored in Arctic and boreal ecosystems, which are under the threat of remineralization from increased microbial activity due to global warming [1, 2]. Microbes play a vital role in nutrient cycling by decomposing soil organic carbon and raising greenhouse gas emissions [3]. Arctic tundra soils harbour diverse microbial communities dominated by members of the phyla *Actinomycetota*, *Acidobacteriota*, *Pseudomonadota*, *Verrucomicrobiota*, and *Bacteroidota* [4–7]. Among these, the *Bacteroidota* are Gram-negative bacteria found in diverse habitats such as soil, freshwater, ocean, plants, and the gastrointestinal tract of animals [8, 9]. Numerous species within the *Bacteroidota* are recognized for their ability to break down complex organic material [8]. The distribution and activity of different members of the tundra soil microbiota [10–14] is associated with soil organic matter breakdown and nutrient cycling in these environments. Increased biodegradation of sequestered carbon in these regions is expected to be a significant contributor to greenhouse gas emissions [15, 16].

The genus *Mucilaginibacter* was proposed in 2007 with the type-species *Mucilaginibacter paludis*, isolated from a Siberian peat bog and named because of its mucus-producing nature [17]. The genus belongs to the family *Sphingobacteriaceae* in the phylum *Bacteroidota* [17]. The genus currently comprises over 80 species with validly published names (https://lpsn.dsmz.de/) [18]. Members of the genus have been isolated from diverse habitats, including aquatic ecosystems, glaciers, soil, plants, and peatlands [11, 19–22]. Several cold-adapted strains affiliated with the genus *Mucilaginibacter* have previously been isolated from the Arctic tundra soils [11]. Members of *Mucilaginibacter* play a vital role in the degradation of complex carbon. Their abundance in environments rich in soil organic matter, such as tundra soils, suggests their role in carbon cycling [17]. Moreover, *Mucilaginibacter* strains produce extracellular polymeric substances, thereby making them well adapted to fluctuating extreme conditions of tundra soils [23]. Though members of the genus *Mucilaginibacter* are present in diverse habitats, little is known about what shapes their taxonomic diversity and their ecological roles and niches in these habitats. The current study reports on five new species of *Mucilaginibacter* isolated from the Arctic tundra heath soils of northern Finland. Moreover, to understand the ecological significance, abundance, and diversity of *Mucilaginibacter* strains in the tundra soils, the genomes of these strains are compared with the genomes of *Mucilaginibacter* spp. isolated from other habitats. We also examined the distribution of the *Mucilaginibacter* in a set of tundra heath soils from which the novel species were isolated. This study expands our understanding of the diversity, ecological significance, and role of *Mucilaginibacter* strains in complex carbon degradation and cycling in Arctic tundra soils.

## Materials and methods

### Strain isolation

*Mucilaginibacter* strains were isolated from tundra soil samples collected from the Kilpisjärvi region, Finland (69°01′N, 20°50′E). Strains E4BP6, X5P1, and X4EP1 were isolated from soil sampled in July 2012 from the north side of Mt. Pikku-Malla in Malla Nature Reserve; strain SP1R1 was isolated from the north side of Mt. Saana; and strain FT3.2 was isolated from a soil incubation experiment after three freeze–thaw cycles of soil sampled from Mt. Pikku-Malla [10]. Isolation and characterization of strains *Mucilaginibacter mallensis* MP1X4, *Mucilaginibacter lappiensis* ANJLi2, and MP601 are described in [11]. Several carbon substrates were tested during the isolation and different strains were cultivated with different media. Strains FT3.2 and SP1R1 were isolated using R2A (pH 7). Strains X5P1 and X4EP1 were isolated using a mixture of carboxymethyl cellulose, xylan, pectin, and starch (each at 0.25 gl^−1^) in VL55 mineral salt medium [24] amended with yeast extract (0.1 gl^−1^) and agar (20 gl^−1^) and pH adjusted to 4.5. Strain E4BP6 was isolated on a medium containing soil and *Empetrum* extract. *Empetrum* extract was prepared from 28 g of crowberry (*Empetrum nigrum*) leaves in 400 ml of water by shaking (220 rpm) for 30 min. Soil extract was prepared by shaking 20 g of soil for 6 h, after which it was centrifuged (4000G/5 min), and the extract was decanted and autoclaved for further use. The growth medium contained 100 ml^−1^of *Empetrum* extract, 300-ml^−1^ soil extract, 0.2-gl^−1^ yeast extract, and 250-ml^−1^ VL55 mineral medium. All strains were maintained either on R2A or GY medium at pH 5.5–6.0. GY medium contained glucose (1 gl^−1^) and yeast extract (0.5 gl^−1^) in VL55.

### Analysis of *Mucilaginibacter* community in tundra heath soils

Soil samples were collected from tundra heaths of Mt. Pikku-Malla in Malla Nature Reserve, Kilpisjärvi (69°03′50″N, 20°44′40″E), with differences in topography that dramatically influence snow accumulation. Four plots representing windswept slopes and four plots corresponding to snow-accumulating biotopes were sampled at a depth of <5 cm in February 2013, as described previously [25]. Composite soil samples of five soil cores were taken from each plot, with three subsamples from each composite sample used for DNA extraction with a CTAB-based method [25].

Near full-length bacterial ribosomal RNA (rRNA) operons were amplified from extracted DNA using 16S rRNA-27F and 23S rRNA-2241R primers, <10-ng template DNA, and a high-fidelity Taq polymerase (Biomake Inc., CA, USA; [26]) with PCR conditions and rRNA operon amplicon analysis as described in [27]. Library construction utilized the SQK-LSK108 sequencing kit and sequencing via the Oxford Nanopore MinION (Oxford, UK). The fast5 files were basecalled using Guppy (3.2.0). Raw reads were demultiplexed with Guppy and sized (3700–5000 bp) using Geneious (11.1.5). FastA files were initially screened via MegaBLAST (2.10.0) against the ribosomal RNA operon database (rOPDB; [28]) to determine the raw reads associated with the *Mucilaginibacter* spp. These reads were rescreened against a modified database amended with rRNA operons from the new *Mucilaginibacter* strains described in this study. Best BLAST hits were identified using the following settings: word size, 60; match/mismatch cost, 2/−3; gap open/extend penalties, 0/−4; and e-value, 1 × 10^−10^. Relative abundances of the different *Mucilaginibacter* spp. were calculated from the combined reads of four replicate soil samples, each from the windswept and snow-accumulating plots.

### Phenotypic and Fatty Acid Methyl Ester (FAME) analysis

The assimilation of various carbon sources by *Mucilaginibacter* strains was tested using Biolog PM2A plates (Biolog Inc, Hayward, CA, USA). The isolates were inoculated in the PM2A plates and incubated at 25°C for 7 days. Growth in the PM2A plate wells was observed by measuring the OD at 600 nm and by checking for a change in the redox indicator colour. Growth temperature limits were tested by cultivating the strains on R2A plates (pH 6) for 2 weeks at 2°C–34°C. The effect of pH on growth was evaluated at 20°C by growing the strains in liquid GY medium at pH 4.0–8.0 (in 0.5-pH unit increments) in 96-well microtiter plates.

Cellular fatty acids were analysed from cells grown on R2A agar (pH 6) at 20°C for 3 days. Total fatty acids were methylated as described earlier [29] and analysed by gas chromatography–mass spectrometry (Agilent 6890 Series Gas Chromatography System and a 5973 Mass Selective Detector, Santa Clara, CA, USA) with an HP-5MS column (30 m, 0.25 mm i.d., 0.25-μm film thickness) with helium as the carrier gas. Fatty acid methyl esters were identified by their retention times (equivalent chain length, ECL values) and mass spectra.

### Genome sequencing and assembly generation

DNA was extracted from the isolates using the DNeasy UltraClean Microbial Kit (Qiagen) according to the manufacturer’s instructions. The genomes of the five new *Mucilaginibacter* strains were sequenced using the Oxford Nanopore MinION. The genomic libraries were prepared using the MinION Rapid Sequencing Kit (SQK-RAD004) followed by sequencing on MinION-Mk1C with R9.4 flow cell. The raw pod5 reads were basecalled with Dorado basecaller v0.4.3 in high accuracy mode. All the studied strains also had Illumina short-read sequences publicly available via the JGI Genome Portal (Table S1). The basecalled FASTQ reads of strains (X5P1, E4BP6, X4EP1, SP1R1) were assembled using Trycycler tool v0.5.4 [30]. Conversely, the reads of strain FT3.2 were assembled using Flye assembler v2.9.3 since Trycycler was unable to assemble a complete genome for the strain due to slightly lower read coverage. Trycycler generates assemblies using Flye v2.9.3 [31], Minipolish v0.1.3 [32], and Raven v1.8.3 [33] assemblers at default settings. The Trycycler-generated assemblies were polished using Nanopore and Illumina reads using Medaka v1.11.1, Polypolish v0.5.0 [34] and POLCA tool v4.1.0 [35]. The Flye-generated assembly of the strain FT3.2 was polished according to a previously described method [36]. Briefly, the Flye-generated assembly was polished with Nanopore reads using two rounds of Racon v1.4.3 and one round of Medaka Polisher v1.11.1. Finally, the Nanopore polished assembly was polished with Illumina short reads using Polypolish v0.5.0 and POLCA v4.1.0 tool. The final assembly of all the studied strains was checked for completeness and contamination using CheckM tool v1.2.2 [37] and genome quality using QUAST tool v5.2.0 [38]. Assembly and genome statistics of the *Mucilaginibacter* strains are listed in (Table S2).

### Genome analysis

The genome assembly of all the tundra *Mucilaginibacter* strains were uploaded to the RAST server for annotation using subsystem technology [39–41]. Additionally, the genomes were analysed using DRAM [42] and METABOLIC tool v4.0 [43, 44] to predict metabolic and biogeochemical functional traits. The metabolic tool annotates microbial genomes using KEGG [45], Pfam [46], custom hidden Markov model [47], and TIGRfam databases [48]. The secondary metabolites encoded by the tundra strains were predicted using the antiSMASH v7 tool [49], while the proviral sequences present in the bacterial strains were predicted by the geNomad v1.8.0 tool [50]. The antiphage defence systems in the genomes were evaluated using the Defense Finder web service [51]. Polyphenol metabolism by the isolates was assessed by using the CAMPER tool [52].

### Phylogenetic, phylogenomic, and pangenome analyses

The 16S rRNA gene sequence was extracted from the whole genome assembly of each tundra isolate using the Basic Rapid Ribosomal RNA Predictor (Barrnap v0.9) tool. The extracted 16S rRNA gene sequences were aligned with all *Mucilaginibacter* spp., and a maximum likelihood tree was prepared using MEGA 11 [53] with 1000 bootstrap replications. The phylogenomic analysis of the *Mucilaginibacter* strains was done as described previously [54]. Briefly, UBCG v3 [55] was used for the phylogenomic tree construction of the strains. The UBCG v3 tool extracts the conserved genes from all the strains and prepares the tree using RAxML [56]. The average nucleotide identity (ANI) values between the strains were calculated using the OrthoANI tool [57]. The digital DNA–DNA hybridisation (dDDH) values were evaluated using the genome-to-genome distance calculator [58].

The pangenome analysis of the eight strains isolated from tundra soil with 42 genomes of publicly available *Mucilaginibacter* strains isolated from water, soil, moss, glacier, plant, and rhizosphere samples was done using Anvi’o v8 [59] following previously described methods [60]. A contig database of all the genomes was created, followed by annotation using NCBI-COGs, tRNA-scan, single-copy core gene (SCG)-taxonomy, and KEGG database. The pangenome was calculated using NCBI-BLAST search, and the Markov Cluster algorithm [61] at an inflation value of 6 was used to cluster the amino acids based on sequence similarity. Finally, the core and unique genes and functional enrichment between the strain’s category were computed with anvi-compute-functional-enrichment-in-pan command using COG20-pathways and KEGG-module annotations.

## Results and discussion

### Tundra isolates represent novel species of *Mucilaginibacter*

**Figure 1:**
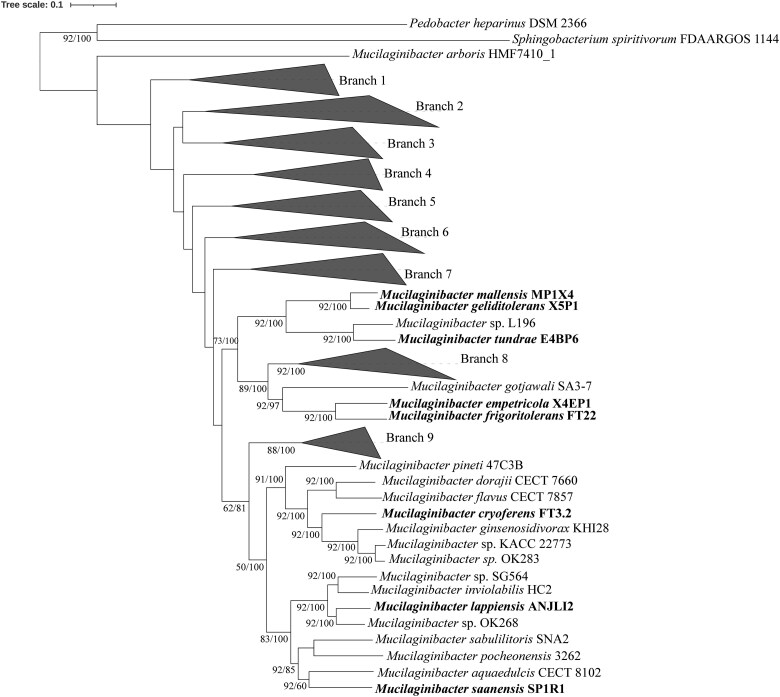
Phylogenomics tree of eight tundra soil isolates and other members of the *Mucilaginibacter* genus. The tree was prepared with the UBCG v3 tool employing RAxML. The value at the branch point represents the gene support index (GSI, indicating the number of genes out of 92 conserved genes supporting the branch point) and the bootstrap value, respectively. 1000 bootstrap replications were used for phylogenomics tree preparation. *Sphingobacterium spiritivorum* FDAARGOS_1144 and *Pedobacter heparinus* DSM 2366 were used as outgroups.

Five bacterial strains were isolated from tundra heath soils. The initial 16S rRNA gene-based phylogenetic analysis denoted that these strains were members of the genus *Mucilaginibacter* (Fig. S1). Further whole genome-based phylogenomic analysis indicated that the Arctic *Mucilaginibacter* strains are distributed across the genus (Fig. 1). The strains E4BP6 and FT3.2 were distant from any described *Mucilaginibacter* spp. In contrast, strain X5P1 clustered with *M. mallensis* MP1X4, strain X4EP1 clustered with *Mucilaginibacter frigoritolerans* FT22 and strain SP1R1 clustered with *M*. *pocheonensis* 3262. The calculated ANI and dDDH values between the tundra strains and their closest relatives from the phylogenomic analysis were all below the threshold value used for species delineation (Table 1). The ANI and dDDH similarities, along with their placements in the phylogenomic tree (Fig. 1), clearly separate the tundra heath isolates from known species. Here we describe five novel species of the genus *Mucilaginibacter* with their respective type strains, for which we propose the names *Mucilaginibacter geliditolerans* sp. nov. X5P1, *Mucilaginibacter tundrae* sp. nov. E4BP6, *Mucilaginibacter empetricola* sp. nov. X4EP1, *Mucilaginibacter saanensis* sp. nov. SP1R1, and *Mucilaginibacter cryoferens* sp. nov. FT3.2. The complete circular phylogenomic tree of all *Mucilaginibacter* spp. is shown in (Fig. S2).

**Table 1:** OrthoANI and dDDH values between the *Mucilaginibacter* strains isolated from the tundra soil and their closest relatives.

Genome pairs	ANI-value	dDDH value
*M. geliditolerans* X5P1 versus *M. mallensis* MP1X4	92	68.5
*M. empetricola* X4EP1 versus *M. frigoritolerans* FT22	81	33
*M. cryoferens* FT3.2 versus *M. dorajii* CECT_7660	80	26.4
*M. saanensis* SP1R1 versus *M. pocheonensis* 3262	79	23.2
*M. tundrae* E4BP6 versus *M. mallensis* MP1X4	76	16.3

Both ANI and dDDH values for the studied strains were below the value used for species delineation, implying that the isolated strains are novel species.

### Novel *Mucilaginibacter* species are polyphenol and complex carbohydrate-degrading specialists

**Figure 2:**
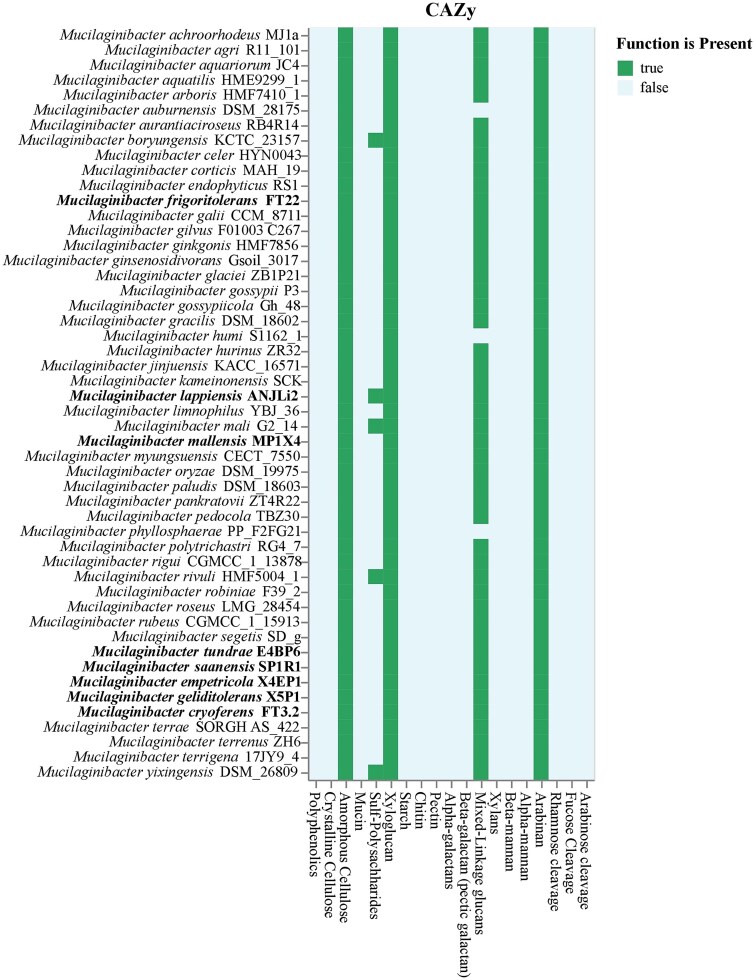
Carbohydrate degradation ability of the members of the genus *Mucilaginibacter* predicted by the CAZy database. The *Mucilaginibacter* strains were able to utilise cellulose, xylose, arabinose, and mixed glycans. The carbohydrate degradation ability is present in all the *Mucilaginibacter* strains irrespective of the isolation source.

The genomic features of the tundra strains analysed using Metabolic and RASTtk tools provided insights into their potential metabolic functions and activities in tundra soils. The genome features were sorted into functional hits (Table S3), indicating that the tundra strains were mainly involved in the degradation of phenolic and other complex carbon compounds, fermentation, and metal reduction. The members of the phylum *Bacteroidota* are known for their ability to degrade complex carbohydrate substrates. Carbon source utilization by the tundra isolates is shown in (Table S4). The genome analysis showed that all the members of the *Mucilaginibacter* genus can degrade carbohydrates, such as cellulose, xyloglucans, mixed linked glucans, and arabinan (Fig. 2). This predicted carbohydrate degradation ability of the various *Mucilaginibacter* strains was not specific to any niche, as they are present in all the strains isolated from different habitats. Tundra soils store substantial amounts of organic carbon in the form of plant litter and soil organic matter that is susceptible to degradation by microbial activity due to rise in temperatures [7]. Previous studies indicated that members of the phylum *Bacteroidota* were more abundant in tundra soils subjected to freeze–thaw cycles [10] and increased at lower temperatures in an incubation experiment [62]. Moreover, the higher abundance of *Bacteroidota* under light reindeer grazing [62] and lower N availability [7] suggests that they are well adapted to the nitrogen-limited tundra heaths dominated by ericaceous shrub vegetation that produces complex, polyphenol-rich plant, and fungal biomass in soil [63]. Several previous studies indicated an acceleration of litter decomposition in the tundra ecosystem due to increased microbial activity [64–66]. The presence of *Mucilaginibacter* strains in tundra sites suggests a role in litter decomposition and carbon recycling. Moreover, functional hits for fermentation processes were also observed in the tundra isolates. Many *Mucilaginibacter* strains are facultative anaerobes [67–70] and gain energy from fermentation under anaerobic conditions.

**Figure 3:**
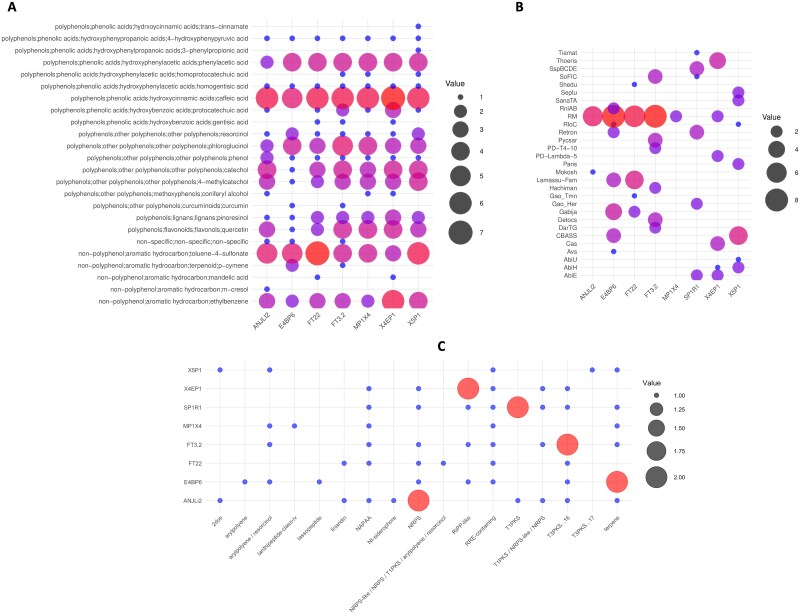
Polyphenol degradation enzymes (A), antiphage defence systems (B), and secondary metabolite synthesis (C) gene clusters present in *Mucilaginibacter* strains. The novel *Mucilaginibacter* strains show the presence of genes related to polyphenol degradation. Antiphage-related genes were also found in the novel strains, implying the presence of phages in the tundra ecosystem. Additionally, novel tundra isolates contain secondary metabolite gene clusters having antimicrobial properties.

Tundra soil habitats are characterized by high plant-derived phenolic compounds [71]. In Fennoscandian tundra ecosystems, shrub-dominated vegetation has been shown to correlate positively with a higher proportion of (poly)phenolic compounds in soils [63, 72]. As the shrub-dominated tundra contains ample amounts of polyphenols and related compounds, the *Mucilaginibacter* strains were evaluated for their ability to metabolize polyphenols using the CAMPER tool. The analysis indicated that the strains harbour enzymes for the degradation of aromatic hydrocarbons, flavonoids, lignans, phenolic acids, and other polyphenols (Fig. 3A). The occurrence of genes involved in the degradation of polyphenols and other aromatic compounds in the *Mucilaginibacter* strains suggests their role in the degradation of plant phenolic compounds in the ericaceous shrub-dominated tundra soils.

### Novel *Mucilaginibacter* species are well-adapted to cold tundra ecosystems and harbour prophages and antiphage systems

Annotation with the RASTtk toolkit provided further insights into the metabolic adaptations of the *Mucilaginibacter* strains to the tundra soil habitat. The RAST annotation showed that the tundra strains harboured genes involved in osmotic, periplasmic, and cold stress responses (Table S5). Genes involved in DNA repair were also prominent in the studied strains (Table S5). The tundra ecosystem is an extreme environment characterized by seasonal changes in temperature, including freeze–thaw cycles, that lead to osmotic and cold stress. Microbes produce various biomolecules for their protection to withstand the extreme conditions of their environment. The presence of osmotic, periplasmic, and cold stress response proteins in the genomes of *Mucilaginibacter* strains indicates the adaptational potential of the strains to their environment.

Bacteriophages can affect bacterial populations and community diversity by mediating horizontal gene transfer, altering the competitiveness among bacterial strains, and maintaining bacterial diversity [73]. In addition to promoting bacterial speciation by horizontal gene transfer events by prophages, bacteriophages also decrease speciation by inducing directional selection of the bacterial cells [74]. The genomes of the novel *Mucilaginibacter* strains (ANJLi2, E4BP6, SP1R1, X4EP1) contain many proviral sequences (Table S6). The presence of these prophage regions in the genomes suggests that they might be helpful in horizontal gene transfer and facilitate the adaptive evolution of strains harbouring them. Since prophage regions were detected in some *Mucilaginibacter* genomes, antiphage defence systems were also explored in the strains. The tundra strains harbour antiphage defence genes that may protect them from phage attacks, promoting speciation events by limiting phage infection that lowers the speciation in bacteria (Fig. 3B).

### Novel *Mucilaginibacter* species are highly suited to nitrogen-deficient tundra soils and produce a wide array of secondary metabolites

The Arctic tundra ecosystems are nitrogen-limited with low inorganic nitrogen concentrations, restricting microbial growth [75]. Microbes adapt and survive in these nitrogen-limiting environments by developing systems to acquire and transport scarce nitrogen into the cell. Nitrogen assimilation genes were therefore investigated in the *Mucilaginibacter* strains isolated from nitrogen-limited tundra soil sites (Table S7). Genes for assimilatory nitrite and nitrate reduction that convert the inorganic nitrate/nitrite into usable organic nitrogen compound, i.e. ammonia, were present. Moreover, the transporters for nitrate/nitrite were also present in the genomes. Additionally, ammonia uptake, transport, and assimilation genes were found in the *Mucilaginibacter* genomes (Table S7). The *Mucilaginibacter* strains appeared to be capable of assimilating both inorganic and organic forms of nitrogen from the environment.

The tundra soil *Mucilaginibacter* strains harbour gene clusters for the synthesis of various secondary metabolites (Fig. 3C). Microbes synthesize secondary metabolites that primarily function as defence mechanisms and inhibit the growth of other microbes, thereby allowing them to compete for resources in their surroundings [76]. They also help their adaptation to enhance their survival and perform other functions, such as communication and establishing symbiotic relations with other microbes [77]. The metabolites synthesized by the predicted gene clusters of the *Mucilaginibacter* strains function as antimicrobial agents, inhibiting the growth of other bacteria, viruses, and fungal strains. They also help in communications, thereby likely providing a competitive advantage of the *Mucilaginibacter* spp. in these tundra habitats.

**Figure 4:**
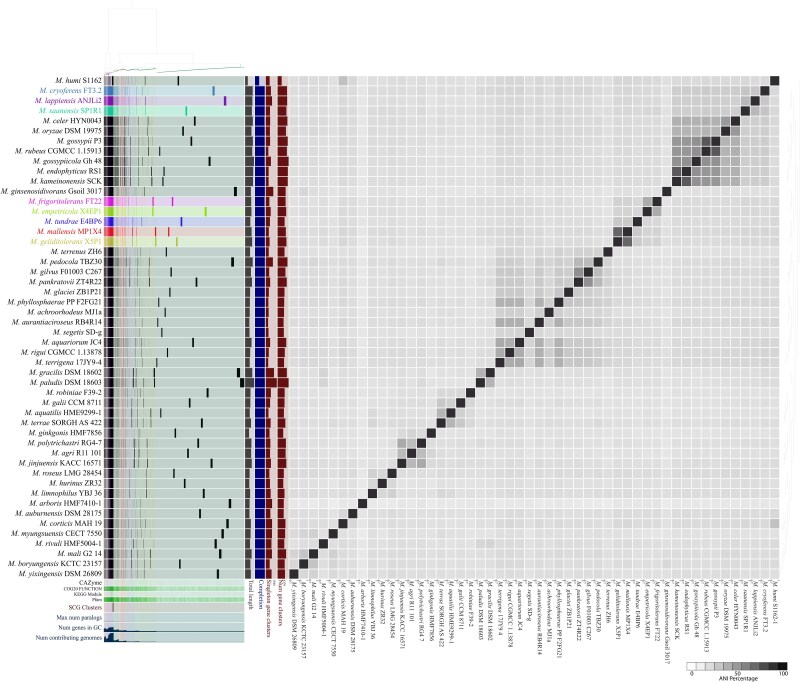
Pangenome analysis of tundra *Mucilaginibacter* strains with other members of the genus isolated from diverse habitats. The heatmap represents the ANI between the strains. The analysis shows that the tundra isolates are separated into two groups based on gene cluster presence and absence. This implies that tundra strains are different from strains isolated from other habitats.

### Comparative pangenome analysis of tundra isolates uncovers distinct and shared functions

Tundra isolates were compared with other *Mucilaginibacter* strains isolated from different habitats. Pangenome analysis of 8 tundra strains with 42 other *Mucilaginibacter* spp. identified 50 667 gene clusters with 241 695 genes in common (Fig. 4). SCG clusters were present in all 50 genomes. The unique genes in the tundra strains are involved in functions such as fatty acid biosynthesis, pyrimidine degradation, and NADH dehydrogenase, as evaluated by COG20 pathway prediction (Table 2). Microbes in the cold tundra ecosystem are subjected to harsh conditions, and various biomolecules need to be synthesized for protection and survival. One of the challenges in cold-temperature habitats is maintaining cell membrane fluidity. Microbes inhabiting cold environments synthesize unsaturated, branched-chain, and shorter acyl-chain fatty acids and incorporate these in the cell membranes to maintain cell fluidity [78–80]. The primary cellular fatty acids of the *Mucilaginibacter* isolates are iso-C15:0, C16:0, C16:1ω7c/iso-C15:0 2-OH (co-elute), iso-C17:1, C16:0 3-OH, and iso-C17:0 3-OH (Table S8). Pyrimidine degradation is useful in microbes as it helps recycle and assimilate nitrogen for growth [81]. As tundra soils are mostly nitrogen deficient, *Mucilaginibacter* strains use nitrogen recycled from pyrimidine degradation for growth. Further, the NADH dehydrogenase synthesizing gene cluster was uniquely present in the tundra strains. There are two types of NADH dehydrogenases present in the bacteria *viz.* NADH-1 enzyme complex and NADH-2 [82]. NADH-1 enzyme complex translocates protons across the cell membrane and oxidizes NADH to NAD^+^, producing energy, while NADH-2 is nonproton-translocating in nature [83]. The tundra strains contain the NADH-1 type enzyme complex in the genome, suggesting their energy generation and survivability capabilities in harsh environments. KEGG module prediction of the unique genes of the tundra strains revealed functions such as aerobactin biosynthesis from lysine, catecholamine biosynthesis, melatonin biosynthesis, and dihydrokalafungin biosynthesis from octaketide (Table 2). Aerobactin is a siderophore that is helpful in the assimilation of iron from the environment and is essential for microbial growth [84]. Iron is a crucial cofactor in various enzymes involved in cellular processes, including respiration, DNA synthesis, and oxidative protection [85]. Catecholamines are essential to bacterial growth by assisting iron utilization [86, 87]. Biosynthesis of the aerobactin siderophore and catecholamine by the tundra *Mucilaginibacter* strains suggest the importance of iron uptake potential for adaptation to the alpine-tundra ecosystem. Melatonin is helpful in the protection of bacterial cells from reactive oxygen species [88, 89], whereas dihydrokalafungin acts as an antimicrobial agent that kills or slows down the growth of microbes [90, 91]. In summary, the tundra soil *Mucilaginibacter* strains contain unique gene clusters that are helpful in the adaptation of the strains to extreme conditions, nitrogen and iron assimilation, energy generation, and growth. Moreover, the genomes of these strains contain genes for complex carbon degradation, response proteins to stressors, polyphenol degradation, biogeochemical cycling, secondary metabolite synthesis helpful for growth and survival, etc., thereby supporting their occurrence in extreme habitats such as tundra soil.

**Table 2:** Unique gene clusters and their functions predicted by COG20 and KEGG modules in the tundra *Mucilaginibacter* isolates.

COG20 pathway	Enrichment score	Adjusted *q*-value	Accession	Gene cluster IDs
Fatty acid biosynthesis Pyrimidine degradation	14.87	0.25	COG0236, COG2070, COG3321, COG4221	GC_00010161
NADH dehydrogenase	4.76	1	COG0649, COG0852	GC_00049369
Na^+^-translocating Fd:NADH oxidoreductase	4.67	1	COG4658	GC_00000098, GC_00010394
Phospholipid biosynthesis Ubiquinone biosynthesis	13.24	0.42	COG0204, COG2227, COG4258	GC_00006052
Asparagine biosynthesis	9.89	1	COG0367	GC_00002406, GC_00005847, GC_00008361, GC_00008958, GC_00009897, GC_00016362, GC_00019377, GC_00020793, GC_00023172, GC_00023208, GC_00032842, GC_00042418, GC_00042651, GC_00044058, GC_00046196, GC_00047875
KEGG module				
Aerobactin biosynthesis, lysine = > aerobactin	9.71	0.84	M00918	GC_00021183
Catecholamine biosynthesis, tyrosine = > dopamine = > noradrenaline = > adrenaline, Melatonin biosynthesis, Tryptophan = > serotonin = > melatonin	9.71	0.84	M00042, M00037, M00936	GC_00017643
Dihydrokalafungin biosynthesis, octaketide = > dihydrokalafungin	10.35	0.82	M00779	GC_00010089, GC_00026961

The comparative genome analysis also revealed the core, shared genes and functions present in all the analysed *Mucilaginibacter* spp. (Fig. S3). The genes for amino acid biosynthesis, like arginine, aromatic amino acid, glutamine, histidine, isoleucine, leucine, valine, lysine, and serine were present in all the *Mucilaginibacter* spp. Moreover, the genes for central carbon metabolism—like glycolysis, pyruvate oxidation, TCA cycle, pentose phosphate pathway, and gluconeogenesis—were observed in all the strains. Additionally, other metabolic functions, such as FoF1-type ATP synthase, biotin, folate, heme, isoprenoid, lipoate, menaquinone, NAD, phospholipid, purine and pyrimidine, riboflavin, thiamine, and ubiquinone biosynthesis were common in all the strains. The detection of cofactor and coenzyme synthesis genes, along with central carbon and amino acid metabolism, in the core genome of *Mucilaginibacter* spp. indicates their capability to effectively utilise resources for growth, adaptation, and survival. This reveals that the members of the genus *Mucilaginibacte*r are well equipped to adapt and grow across various environments, as is also evident from their cultivation from a wide variety of habitats.

### Ecological context of *Mucilaginibacter* community in tundra heath soils

**Figure 5:**
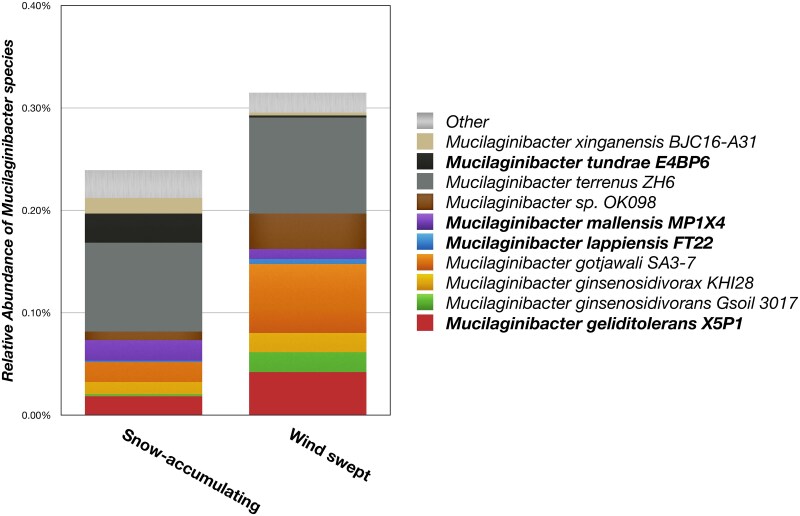
Relative abundance of detected *Mucilaginibacter* species in soils of windswept and snow-accumulating tundra heath plots of Mt. Pikku-Malla. rRNA operon reads from the *Bacteroidota* represented ~1.7% and *Mucilaginibacter* spp. ~0.25%–0.32% of the total bacterial reads. Data present the combined reads of four replicate soil samples, each from the windswept and snow-accumulating plots.

We examined the distribution of the *Mucilaginibacter* spp. in a set of snow-accumulating and windswept tundra heath soils of Malla Nature Reserve, including soil plots from which the novel species were isolated. At this site, variation in topology results in depressions sheltered from the winds with high snow accumulation (up to ≥1 m), contrasting with windswept areas that remain essentially snow-free throughout the winter. This leads to distinctly different soil temperature profiles and differences in the amplitude of annual temperature variation [25]. The soil bacterial communities were assessed by rRNA operon profiling with the Oxford Nanopore MinION, enabling strain-specific identification of community members. Overall, rRNA operon reads from the *Bacteroidota* represented 1.7% of the total bacterial reads from these tundra samples. The *Mucilaginibacter* reads represented ~0.25% of the rRNA operon reads in the snow-accumulating soils and ~0.32% in the windswept soils. Several different *Mucilaginibacter* spp. were detected, including *M. tundrae*, *M. mallensis*, *M. lappiensis*, and *M. geliditolerans*, which had all been cultivated from these soils (Fig. 5). Snow cover, reindeer grazing and the linked vegetation shifts and soil C and N dynamics may be the important microclimatic drivers of bacterial communities. Diverse *Mucilaginibacter* spp. are ubiquitous in acidic Arctic tundra and sub-Arctic Forest soils. The Kilpisjärvi region has representative tundra vegetation dominated by dwarf shrub-rich *Empetrum* heaths over acidic soils or forb- and graminoid-rich *Dryas* heaths over nonacidic soils [10, 72, 92, 93]. These soils are well-aerated and rich in organic carbon, harbouring an abundant and diverse aerobic heterotrophic microbiota.

## Conclusion

Here, we describe five new species of the *Mucilaginibacter* genus isolated from Artic tundra heath soil. The genomic analysis provided insight into their carbon degradation potential, adaptation to extreme conditions, and ecology in their tundra soil habitat. The study shows that the strains were capable of degrading a variety of polysaccharides and polyphenols and contained response proteins for cold, osmotic, and periplasmic stress. The strains harbour genes for carbon cycling and nitrogen assimilation by nitrite ammonification and pyrimidine degradation. Further, the genomes contain unique genes for the biosynthesis of fatty acids required for membrane integrity, enzymes for energy generation, and secondary metabolites for growth that explain the abundance and diversity of *Mucilaginibacter* species in tundra soils. The genomic study provides insights into the ecosystem functions of *Mucilaginibacter* species in tundra soil and points out the role of these microbes in carbon degradation and releasing greenhouse gases from stored organic matter.

### Description of *Mucilaginibacter geliditolerans* sp. nov.

*Mucilaginibacter geliditolerans* (ge.li.di.to’le.rans. L. masc. adj. *gelidus*, cold; L. pres. part. *tolerans*, tolerating, enduring; N.L. masc. part. adj. *geliditolerans*, cold-tolerating).

Cells are Gram-negative, nonmotile, aerobic rods. Colonies are pale yellow and mucoid when grown on R2A agar. Growth occurs at 2°C–32°C and pH 4.5–7.0. The major cellular fatty acids are iso-C15:0, C16:0, C16:1ω7c/iso-C15:0 2-OH (co-elute), iso-C17 : 0 3-OH, and iso-C17:1. The DNA G + C content determined from the genome sequence of the type strain is 41.27%. The type strain is X5P1^T^ (= DSMZ 119435 = HAMBI 3824) isolated from tundra soil in Malla Nature Reserve, Kilpisjärvi, Finland (69°01′N, 20°50′E). NCBI accession numbers for the 16S rRNA gene sequence and the draft genome sequence of the type strain are PQ453000 and CP183230, respectively.

### Description of *Mucilaginibacter tundrae* sp. nov.

*Mucilaginibacter tundrae* (tun’drae. N.L. gen. fem. n. *tundrae*, from the tundra biome).

Cells are Gram-negative, nonmotile, aerobic rods. Colonies are yellow and smooth when grown on R2A agar. Growth occurs at 2°C–34°C and pH 4.0–6.5. The major cellular fatty acids are iso-C15:0, C16:0, C16:1ω7c/iso-C15:0 2-OH (co-elute), iso-C17 : 0 3-OH, and iso-C17:1. The DNA G + C content determined from the genome sequence of the type strain is 39.99%. The type strain is E4BP6^T^ (= DSMZ 119436 = HAMBI 3826) isolated from tundra soil in Malla Nature Reserve, Kilpisjärvi, Finland (69°01′N, 20°50′E). NCBI accession numbers for the 16S rRNA gene sequence and the draft genome sequence of the type strain are PQ452956 and CP183227, respectively.

### Description of *Mucilaginibacter empetricola* sp. nov.

*Mucilaginibacter empetricola* (em.pe.tri’co.la. L. suff. -cola (from L. n. *incola*), inhabitant; N.L. neut. n. *Empetrum*, referring to the plant genus *Empetrum*; N.L. n. *empetricola*, inhabiting tundra heath soil dominated by the plant *E. nigrum ssp. hermaphroditum*).

Cells are Gram-negative, nonmotile, aerobic rods. Colonies are yellow and smooth when grown on R2A agar. Growth occurs at 2°C–34°C and pH 4.5–6.5. The major cellular fatty acids are iso-C15:0, C16:0, C16:1ω7c/iso-C15:0 2-OH (co-elute), iso-C17 : 0 3-OH, and iso-C17:1. The DNA G + C content determined from the genome sequence of the type strain is 40.60%. The type strain is X4EP1^T^ (= DSMZ 119437 = HAMBI 3825) isolated from *E. nigrum* rhizosphere soil from Malla Nature Reserve, Kilpisjärvi, Finland (69°01′N, 20°50′E). NCBI accession numbers for the 16S rRNA gene sequence and the draft genome sequence of the type strain are PQ452973 and CP183229, respectively.

### Description of *Mucilaginibacter saanensis* sp. nov.

*Mucilaginibacter saanensis* (sa.a.nen’sis. N.L. masc. Adj. *saanensis*, pertaining to Mt. Saana in Kilpisjärvi, Finland).

Cells are Gram-negative, nonmotile, aerobic rods. Colonies are pale pink and smooth when grown on R2A agar. Growth occurs at 2°C–32°C and pH 4.5–8. The major cellular fatty acids are iso-C15:0, C16:0, C16:1ω7c/iso-C15:0 2-OH (co-elute), and iso-C17 : 0 3-OH. The DNA G + C content determined from the genome sequence of the type strain is 41.74%. The type strain is SP1R1^T^ (= DSMZ 119438 = HAMBI 3819) isolated from tundra soil on Mount Saana, Kilpisjärvi, Finland (69°01′N, 20°50′E). NCBI accession numbers for the 16S rRNA gene sequence and the draft genome sequence of the type strain are PQ452957 and CP183226, respectively.

### Description of *Mucilaginibacter cryoferens* sp. nov.

*Mucilaginibacter cryoferens* (cry.o.fer.ens. Gr. neut. n. kryos, cold; L. pres. part. *ferens*, to endure; N.L. masc. part. adj. *Cryoferens*, cold-enduring).

Cells are Gram-negative, nonmotile, aerobic rods. Colonies are pale yellow and smooth when grown on GR2A agar. Growth occurs at 2°C–32°C and pH 4.5–8.0. The major cellular fatty acids are iso-C15:0, C16:0, C16:1ω7c/iso-C15:0 2-OH (co-elute), and iso-C17 : 0 3-OH. The DNA G + C content determined from the genome sequence of the type strain is 42.08%. The type strain is FT3.2^T^ (= DSMZ 119439 = HAMBI 3818) isolated from tundra soil in Malla Nature Reserve, Kilpisjärvi, Finland (69°01′N, 20°50′E) after multiple freeze–thaw cycles. NCBI accession numbers for the 16S rRNA gene sequence and the draft genome sequence of the type strain are PQ452958 and CP183228, respectively.

## Supplementary Material

Kumar_et_al_Mucilaginibacter_species_Supplementary_Data

## Data Availability

Type strains are deposited in the German Collection of Microorganisms and Cell Cultures (DSMAZ) and University of Helsinki HAMBI Culture Collection. The NCBI accession numbers for the newly assembled *Mucilaginibacter* genomes are CP183226-CP183230. Accession numbers for 16S rRNA genes are PQ453000, PQ452956, PQ452973, PQ452957, and PQ452958. Accession numbers for rRNA operons are PV018880-PV018893. IMG submission IDs for genomes are 8122391181, 8122369792, 8122385834, 8122374309, and 8122379841. The rRNA operon reads from Malla Nature Reserve soil samples are available in BioProject ID PRJNA1093128.
