# Supplementary material for: Genome analysis reveals diverse novel psychrotolerant Mucilaginibacter species in Arctic tundra soils
Source: ISME Commun. 2025 Apr 25;5(1):ycaf071. doi: 10.1093/ismeco/ycaf071 (PMC12074574; doi:10.1093/ismeco/ycaf071)
Supplement: Kumar_et_al_Mucilaginibacter_species_Supplementary_Data [file kumar_et_al_mucilaginibacter_species_supplementary_data.pdf]

SUPPLEMENTARY DATA

Genome analysis reveals diverse novel psychrotolerant *Mucilaginibacter* species in Arctic tundra soils

Anil Kumar [1,2], Minna K. Männistö [3], Marika Pätsi [4], Lee J. Kerkhof [2], Max M. Häggblom [1]

[1] Department of Biochemistry and Microbiology and [2] Department of Marine and Coastal Sciences, Rutgers University, New Brunswick, NJ 08901, USA

[3] Natural Resources Institute Finland, FI-96200 Rovaniemi, Finland

[4] Natural Resources Institute Finland, FI- 90570 Oulu, Finland

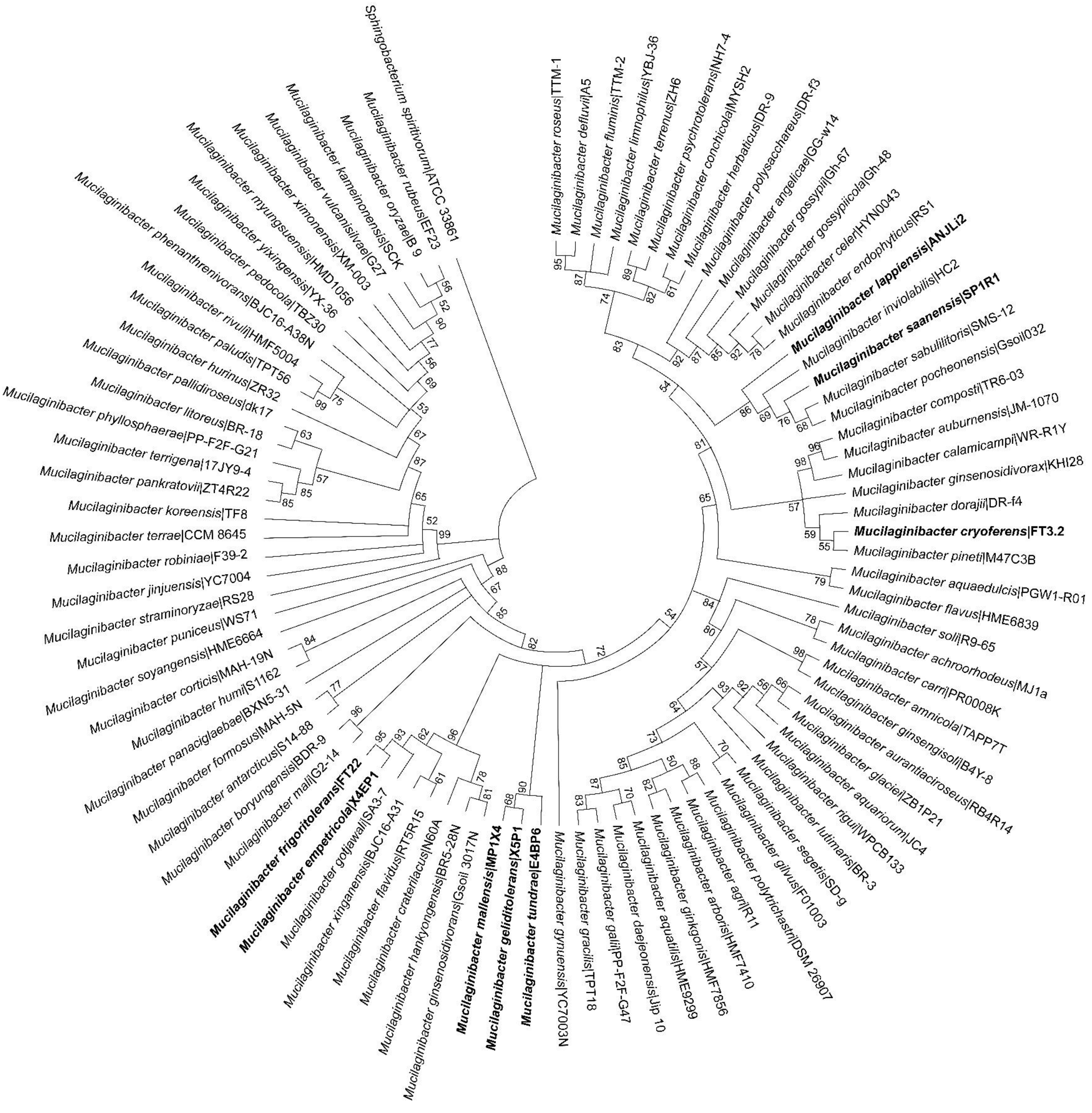

Fig. S1: 16S rRNA-based, maximum likelihood, phylogenetic tree of the *Mucilaginibacter* genus showing the placement of eight strains isolated from Kilpisjärvi region tundra soil (highlighted in bold). *Sphingobacterium spiritivorum* ATCC 33861 was used as an outgroup. The values at the branch point indicate the bootstrap values of 1000 replications; values below 50 are not shown. The tree shows the separation of tundra strains from other described species of the genus *Mucilaginibacter.*

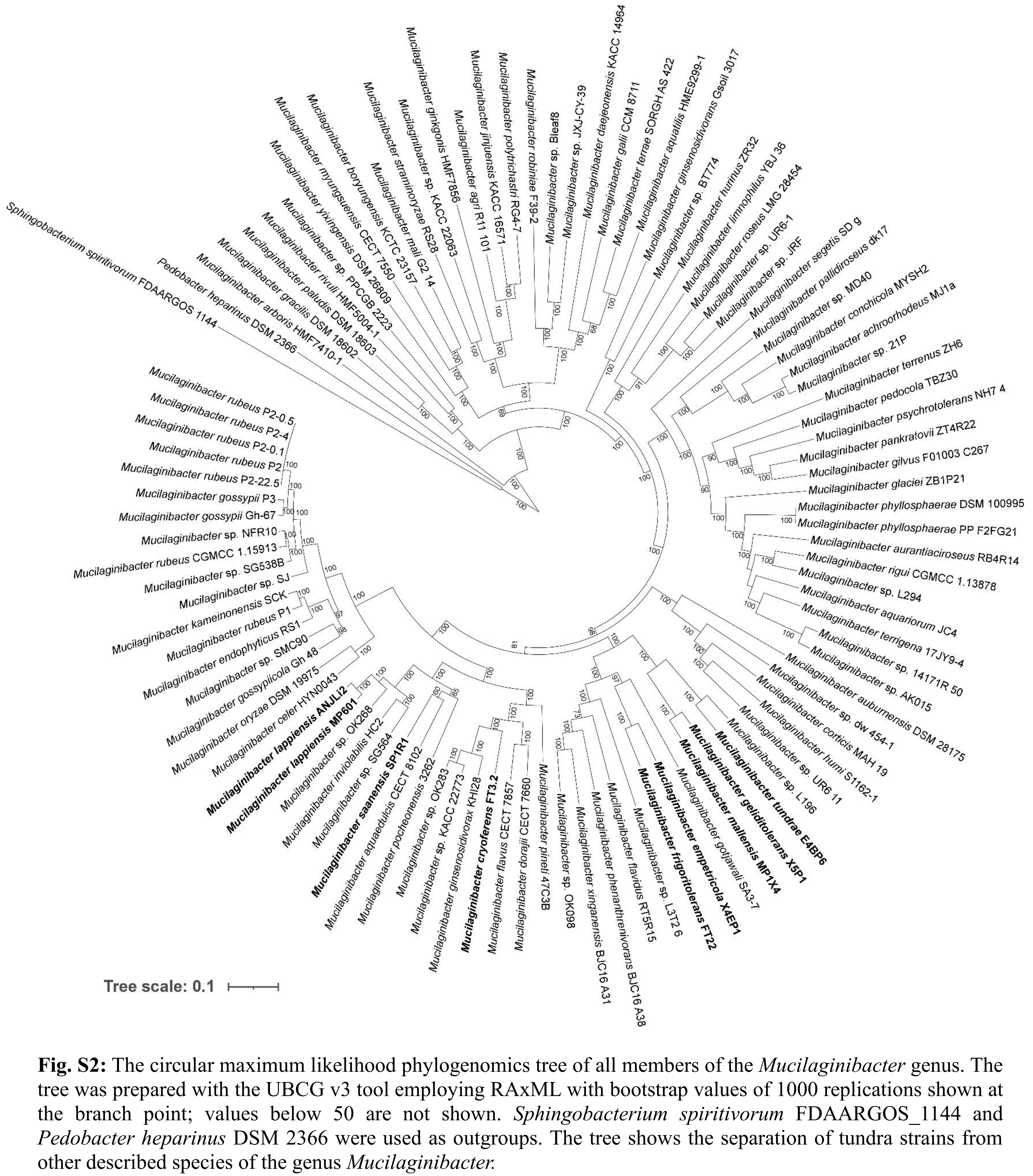

Fig. S2: The circular maximum likelihood phylogenomics tree of all members of the *Mucilaginibacter* genus. The tree was prepared with the UBCG v3 tool employing RAxML with bootstrap values of 1000 replications shown at the branch point; values below 50 are not shown. *Sphingobacterium spiritivorum* FDAARGOS_1144 and *Pedobacter heparinus* DSM 2366 were used as outgroups. The tree shows the separation of tundra strains from other described species of the genus *Mucilaginibacter.*

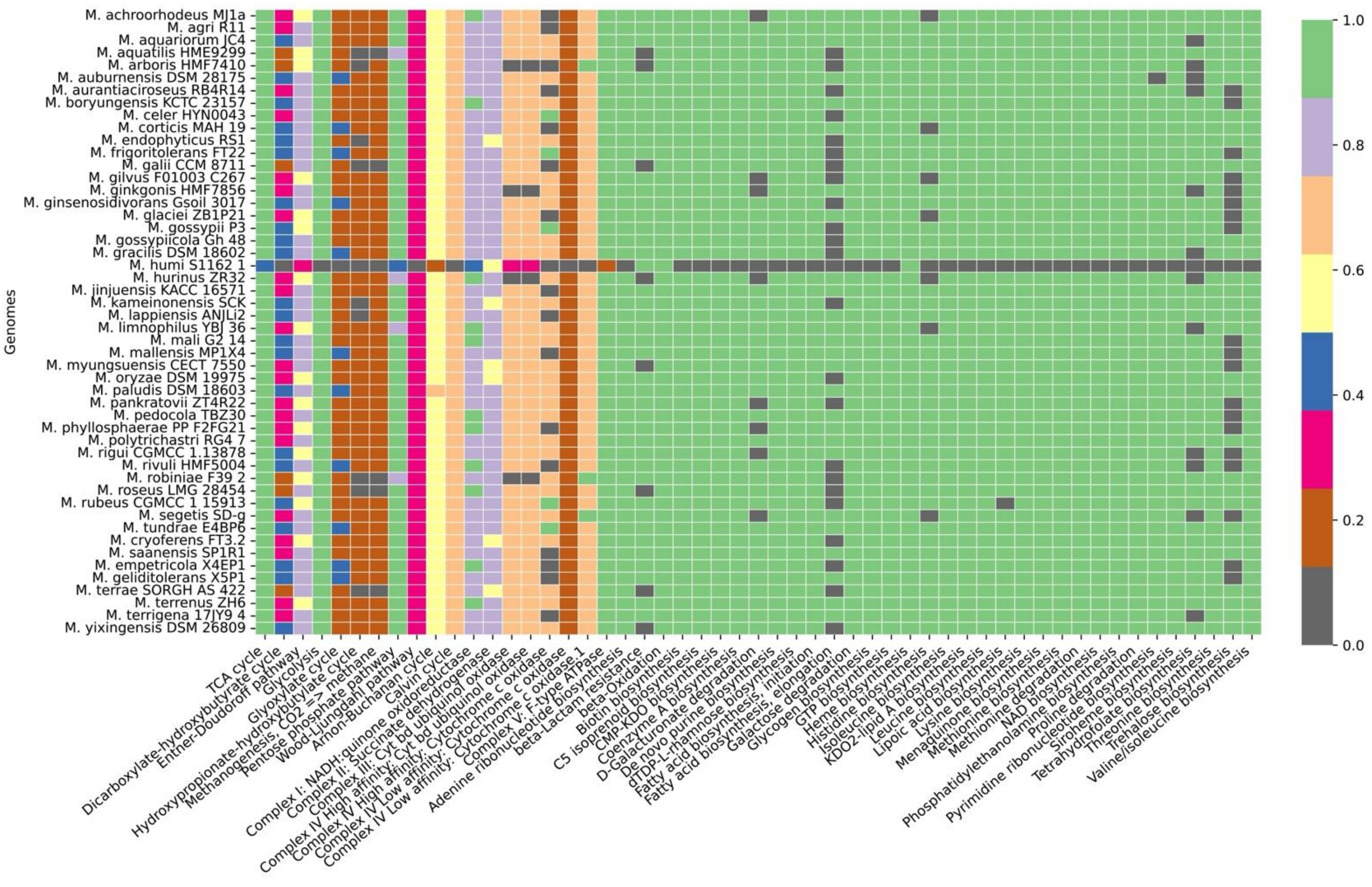

Fig. S3: The core or shared metabolic functions present in all the *Mucilaginibacter* strains. The shared functions include central carbon metabolism, amino acids metabolism, energy generation pathways, lipid metabolism, and multidrug resistance.

Table S1: Illumina sequencing reads and project information of tundra-isolated *Mucilaginibacter* strains. The table shows the old and new IMG IDs of the sequencing project, as well as the NCBI genome and 16S rRNA gene assertions for the new assembly.

Strain name	IMG ID (Illumina)	IMG ID (Hybrid)	NCBI Genome assembly (Hybrid)	16S rRNA
Mucilaginibacter geliditolerans X5P1	2849165822	8122391181	CP183230.1	PQ453000
Mucilaginibacter tundrae E4BP6	2849171336	8122369792	CP183227.1	PQ452956
Mucilaginibacter empetricola X4EP1	2849193241	8122385834	CP183229.1	PQ452973
Mucilaginibacter saanensis SP1R1	2849175832	8122374309	CP183226.1	PQ452957
Mucilaginibacter cryoferens FT3.2	2849317864	8122379841	CP183228.1	PQ452958

Table S2: Assembly and genome statistics of *Mucilaginibacter* strains isolated from tundra sites. The Nanopore and Illumina-based sequences allowed for the complete trycycler and flye hybrid genome assembly of all the strains. The genome size, number of genes, assembly completion & contamination levels were evaluated using the CheckM tool, and genome statistics were evaluated using QUAST.

**Genome name**	**E4BP6**	**FT3.2**	**SP1R1**	**X4EP1**	**X5P1**
Completeness	97.62	97.46	97.46	97.94	97.62
Contamination	1.19	0.48	1.35	0.24	1.19
Size	4864841	7054601	6304161	5882492	6530971
DNA GC%	39.99	42.08	41.74	40.60	41.27
Protein coding genes	4442	5901	5447	5270	5460
CRISPR Arrays	-	1	1	1	-
rRNA genes	6	15	9	6	9
16S rRNA genes	2	5	3	2	3

Table S3: The different functions performed by tundra isolates. The analysis was performed by using the Metabolic toolkit. The tundra *Mucilaginibacter* strains mainly had the potential for complex carbon degradation, aromatics degradation, and metal reduction functions.

Category	Function	Gene abbreviation	ANJLi2	FT22	E4BP6	FT3.2	MP1X4	SP1R1	X4EP1	X5P1
Ethanol fermentation	Ethanol fermentation	acetate => acetaldehyde	Present	Present	Absent	Present	Present	Present	Absent	Present
Aromatics degradation	Phenol => Benzoyl-CoA	ubiX‖bsdC	Present	Present	Present	Present	Present	Present	Present	Present
Complex carbon degradation	Cellulose degrading	cellulase	Present	Present	Present	Present	Present	Present	Present	Present
Complex carbon degradation	Cellulose degrading	beta-glucosidase	Present	Present	Present	Present	Present	Present	Present	Present
Complex carbon degradation	Hemicullulose debranching	arabinosidase	Present	Present	Present	Present	Present	Present	Present	Present
Complex carbon degradation	Hemicullulose debranching	beta-glucuronidase	Absent	Absent	Absent	Present	Absent	Present	Absent	Absent
Complex carbon degradation	Hemicullulose debranching	alpha-L-rhamnosidase	Present	Present	Present	Present	Present	Present	Present	Present
Complex carbon degradation	Endohemicellulases	mannan endo-1,4-beta-mannosidase	Present	Present	Present	Present	Present	Present	Present	Present
Complex carbon degradation	Endohemicellulases	alpha-D-xyloside xylohydrolase	Present	Present	Present	Present	Present	Present	Present	Present
Complex carbon degradation	Other oligosaccharide degrading	beta-xylosidase	Present	Present	Present	Present	Present	Present	Present	Present
Complex carbon degradation	Other oligosaccharide degrading	beta-mannosidase	Absent	Present	Present	Absent	Absent	Absent	Present	Absent
Complex carbon degradation	Other oligosaccharide degrading	beta-galactosidase	Present	Present	Present	Present	Present	Present	Present	Present
Complex carbon degradation	Amylolytic enzymes	isoamylase	Present	Absent	Present	Present	Absent	Present	Absent	Absent
Fermentation	Acetogenesis	acdA‖ack‖pta	Present	Present	Present	Present	Present	Present	Present	Present
Fermentation	Acetate to acetyl-CoA	acs	Present	Present	Present	Present	Present	Present	Present	Present
C1 metabolism	Formaldehyde oxidation	fdhA‖fghA‖frmA‖mycoS_dep_FDH‖fae	Absent	Absent	Absent	Present	Absent	Present	Absent	Absent
C1 metabolism	Aerobic CO oxidation	coxS‖coxM‖coxL	Present	Absent	Absent	Present	Absent	Present	Absent	Absent
Nitrogen cycling	Nitrite reduction to ammonia	nrfADH‖nirBD	Absent	Absent	Absent	Present	Present	Present	Present	Present

Oxidative phosphorylation	Complex I (NADH-quinone oxidoreductase)	nuoABC	Present	Present	Present	Present	Present	Present	Present	Present
Oxidative phosphorylation	Complex II (Succinate dehydrogenase/Fumarate reductase)	sdhCD	Present	Present	Present	Present	Present	Present	Present	Present
Oxidative phosphorylation	Complex V (F-type H+-transporting ATPase)	atpAD (F-type)	Present	Present	Present	Present	Present	Present	Present	Present
Oxygen metabolism (Oxidative phosphorylation Complex IV)	Oxygen metabolism - cytochrome c oxidase, cbb3-type	ccoNOP	Absent	Present	Present	Present	Absent	Absent	Absent	Absent
Oxygen metabolism (Oxidative phosphorylation Complex IV)	Oxygen metabolism - cytochrome (quinone) oxidase, bd type	cydAB	Present	Present	Present	Present	Present	Present	Present	Present
Halogenated compound utilization	Halogenated compounds breakdown	E3.8.1.2‖pcpC‖cprA‖pceA	Absent	Absent	Absent	Absent	Absent	Absent	Present	Absent
Metal reduction	Metal (Iron/Manganese) reduction	Iron reduction series genes	Present	Present	Present	Present	Present	Present	Present	Present

Table S4: Growth conditions and list of carbon sources utilized by novel *Mucilaginibacter* strains. Growth temperature ranges were tested on R2A plates (pH 6) for 2 weeks at 2-34°C. The pH growth range was tested in liquid GY medium at pH 4.0-8.0 (in 0.5 pH unit increments). Carbon source utilization was tested on Biolog PM2A plates.

	Strain				
	**E4BP6**	**SP1R1**	**X4EP1**	**X5P1**	**FT3.2**
**Growth conditions**					
Temperature-range (°C)	2-34	2-32	2-34	2-32	2-32
pH range	4.0-6.5	4.5-8.0	4.5-6.5	4.5-7.0	4.5-8.0
**Assimilation of:**					
2-Deoxy-D- Ribose	+	+	+	w	+
2-HydroxyBenzoic Acid	w	-	w	-	+
2,3-Butanedione	+	+	w	-	+
3-0-ß-D-Galactopyranosyl- D-Arabinose	w	w	-	+	-
3-Hydroxy-2- Butanone	+	w	w	+	+
4-HydroxyBenzoic Acid	+	-	w	+	+
5-Keto-D-Gluconic Acid	+	+	+	+	+
Acetamide	w	w	w	+	+
Amygdalin	-	-	+	+	+
Arbutin	+	-	-	+	+
Butyric Acid	-	w	-	w	+
Capric Acid	-	+	-	-	+
Caproic Acid	-	w	w	+	+
Chondroitin Sulfate C	-	w	w	-	-
Citraconic Acid	+	-	w	+	+
Citramalic Acid	w	-	w	+	+
D-Arabinose	+	-	+	+	+
D-Arabitol	-	-	+	+	+
D-Fucose	w	w	w	w	w
D-Glucosamine	+	-	+	-	+
D-Lactic Acid Methyl Ester	w	w	-	+	+
D-Melezitose	-	-	+	+	+
D-Raffinose	-	-	-	+	+
D-Ribono-1,4- Lactone	+	-	+	+	+
D-Tagatose	+	-	+	+	+
D-Tartaric Acid	w	-	+	+	+
D,L-Carnitine	+	w	w	+	+

Dextrin	w	+	w	+	+
DihydroxyAcetone	+	+	+	+	+
Gelatin	-	w	w	w	w
Gentiobiose	-	-	-	+	-
Glycine	+	-	w	+	+
Glycogen	-	w	w	w	w
Hydroxy-LProline	+	-	+	+	+
Inulin	+	w	-	+	+
Itaconic Acid	+	-	w	+	+
L-Alaninamide	+	+	+	+	+
L-Arginine	w	-	+	+	+
L-Histidine	+	-	w	+	+
L-Lysine	+	w	-	+	+
L-Methionine	w	w	-	w	+
L-Ornithine	w	w	w	+	+
L-PyroglutamicAcid	+	w	w	+	+
L-Sorbose	+	-	+	+	+
L-Tartaric Acid	w	w	-	+	+
Lactitol	+	-	w	+	+
Laminarin	w	w	-	w	w
Malonic Acid	+	+	+	+	+
Maltitol	w	-	+	+	+
Mannan	w	w	-	w	w
Melibionic Acid	+	-	+	+	+
N-Acetyl-D- Galactosamine	-	-	-	w	-
N-Acetyl-L- Glutamic Acid	w	w	+	+	+
Oxalic Acid	w	w	+	+	+
Oxalomalic Acid	+	-	+	+	+
Palatinose	-	w	w	+	+
Pectin	-	w	w	-	w
Putrescine	+	+	w	+	+
Sebacic Acid	+	-	+	+	+
Sedoheptulosan	+	-	w	+	+
Sorbic Acid	+	+	+	-	+
ß-Cyclodextrin	+	+	-	w	w
ß-D-Allose	+	-	w	+	+
ß-Methyl-D- Glucuronic Acid	w	-	w	+	w
Stachyose	w	-	w	+	+

Succinamic Acid	+	+	+	+	+
Turanose	w	-	+	+	+
α-Cyclodextrin	+	+	-	-	w
α-Methyl-D- Glucoside	w	-	+	+	w
γ-Cyclodextrin	+	+	-	-	w

(+) Positive; (-) negative; (w) weakly positive reaction, na (data not available)

Table S5: Stress response proteins present in the tundra soil *Mucilaginibacter* strains. The proteins for osmotic and periplasmic stress were observed in the strains. The proteins for DNA repair and restriction-modification systems were also prominent in the tundra strains.

Subcategory	Subsystem	Role	X5P1	X4EP1	SP1R1	MP1X4	E4BP6	FT22	FT3.2	ANJLI2
DNA Metabolism	Restriction-Modification System	Putative DNA-binding protein in cluster with Type I restriction-modification system	-	-	-	*	-	-	-	-
		Type I restriction-modification system, DNA-methyltransferase subunit M (EC 2.1.1.72)	-	-	-	*	*	*	-	*
		Type I restriction-modification system, restriction subunit R (EC 3.1.21.3)	-	-	-	-	*	-	-	*
		Type III restriction-modification system methylation subunit (EC 2.1.1.72)	-	-	-	-	-	*	-	-
DNA repair	2-phosphoglycolate salvage	Phosphoglycolate phosphatase (EC 3.1.3.18)	*	-	*	*	-	-	-	-
		Putative phosphatase YqaB	*	-	-	*	-	-	-	-
	DNA repair system including RecA, MutS and a hypothetical protein	DNA mismatch repair protein MutS	*	*	*	*	*	*	*	*
		RecA protein	*	*	*	*	*	*	*	*
		Regulatory protein RecX	*	*	*	*	*	*	*	*
	DNA repair, bacterial	A/G-specific adenine glycosylase (EC 3.2.2.-)	*	*	*	*	*	*	*	*
		ADA regulatory protein	*	*	*	*	*	*	*	*
		Alkylated DNA repair protein AlkB	*	*	*	-	*	*	*	*
		DNA polymerase IV (EC 2.7.7.7)	*	*	*	*	*	*	*	*
		DNA recombination protein RmuC	*	*	*	*	*	*	*	*
		DNA repair protein RadA	*	*	*	*	*	*	*	*
		DNA repair protein RecN	*	*	*	*	*	*	*	*
		DNA-cytosine methyltransferase (EC 2.1.1.37)	*	*	*	-	*	*	*	*
		Exodeoxyribonuclease III (EC 3.1.11.2)	*	*	*	*	*	*	*	*
		Methylated-DNA--protein-cysteine methyltransferase (EC 2.1.1.63)	*	*	*	*	*	*	*	*

	RecA protein	*	*	*	*	*	*	*	*
	Single-stranded DNA-binding protein	*	*	*	*	*	*	*	*
	Very-short-patch mismatch repair endonuclease (G-T specific)	-	-	-	-	*	-	-	*
	Error-prone repair protein ImuA	-	-	-	-	*	-	-	*
	Exonuclease SbcC	-	-	-	-	*	-	-	-
	Exonuclease SbcD	-	-	-	-	*	-	-	-
	Endonuclease V (EC 3.1.21.7)	-	-	-	-	-	-	-	*
DNA repair, bacterial MutL-MutS system	DNA mismatch repair protein MutL	*	*	*	*	*	*	*	*
	DNA mismatch repair protein MutS	*	*	*	*	*	*	*	*
	MutS-related protein, family 1	*	*	*	*	*	*	*	*
	Recombination inhibitory protein MutS2	*	*	*	*	*	*	*	*
DNA repair, bacterial photolyase	Deoxyribodipyrimidine photolyase (EC 4.1.99.3)	*	*	*	*	*	*	*	*
	Deoxyribodipyrimidine photolyase, single-strand-specific	*	*	-	*	-	-	-	-
DNA repair, bacterial RecFOR pathway	ATP-dependent DNA helicase RecQ	*	*	*	*	*	*	*	*
	DNA recombination and repair protein RecF	*	*	*	*	*	*	*	*
	DNA recombination and repair protein RecO	*	*	*	*	*	*	*	*
	RecA protein	*	*	*	*	*	*	*	*
	Recombination protein RecR	*	*	*	*	*	*	*	*
	Single-stranded DNA-binding protein	*	*	*	*	*	*	*	*
DNA repair, UvrABC system	Excinuclease ABC subunit A	*	*	*	*	*	*	*	*
	Excinuclease ABC subunit A paralog in greater Bacteroides group	*	*	*	*	*	*	*	*
	Excinuclease ABC subunit B	*	*	*	*	*	*	*	*
	Excinuclease ABC subunit C	*	*	*	*	*	*	*	*
Nonhomologous End-Joining in Bacteria	ATP-dependent DNA ligase (EC 6.5.1.1) clustered with Ku protein, LigD	*	*	*	*	*	*	*	*

		ATP-dependent DNA ligase (EC 6.5.1.1) LigC	-	*	*	-	*	-	-	*
		Ku domain protein	*	*	*	*	*	*	*	*
	RecA and RecX	RecA protein	*	*	*	*	*	*	*	*
		Regulatory protein RecX	*	*	*	*	*	*	*	*
	DNA Repair Base Excision	ATP-dependent DNA ligase (EC 6.5.1.1) clustered with Ku protein, LigD	-	*	*	-	*	-	-	*
		ATP-dependent DNA ligase (EC 6.5.1.1) LigC	-	*	*	-	*	-	-	*
		DNA polymerase I (EC 2.7.7.7)	-	*	*	-	*	-	-	*
		DNA-3-methyladenine glycosylase (EC 3.2.2.20)	-	*	*	-	*	-	-	*
		DNA-3-methyladenine glycosylase II (EC 3.2.2.21)	-	*	*	-	*	-	-	*
		Endonuclease III (EC 4.2.99.18)	-	*	*	-	*	-	-	*
		Ku domain protein	-	*	*	-	*	-	-	*
		Formamidopyrimidine-DNA glycosylase (EC 3.2.2.23)	-	-	*	-	*	-	-	*
Protein folding	Protein chaperones	Chaperone protein DnaJ	*	*	*	*	*	*	*	*
		Chaperone protein DnaK	*	*	*	*	*	*	*	*
		Chaperone protein HscB	*	*	*	*	*	*	*	*
		Chaperone protein HtpG	*	*	*	*	*	*	*	*
		DnaJ-class molecular chaperone CbpA	*	*	*	*	*	*	*	*
		Heat shock protein GrpE	*	*	*	*	*	*	*	*
Detoxification	Uptake of selenate and selenite	DedA protein	*	*	*	*	*	*	*	*
Osmotic stress	Choline and Betaine Uptake and Betaine Biosynthesis	L-proline glycine betaine ABC transport system permease protein ProV (TC 3.A.1.12.1)	-	*	-	-	-	-	-	-
	Osmoregulation	Aquaporin Z	*	*	*	*	-	*	*	*
		Glycerol uptake facilitator protein	*	*	*	*	-	*	*	*
		Outer membrane protein A precursor	*	*	*	*	*	*	*	*
	NADPH:quinone oxidoreductase 2	NADPH:quinone oxidoreductase 2	-	*	*	*	-	-	-	-

	Rubrerythrin	Alkyl hydroperoxide reductase subunit C-like protein	*	*	*	*	*	*	*	*
		Rubredoxin	*	*	-	*	-	-	-	-
		Rubrerythrin	*	*	*	*	*	*	*	*
	Glutathione: Biosynthesis and gamma-glutamyl cycle	Gamma-glutamyltranspeptidase (EC 2.3.2.2)	-	-	-	-	-	-	-	*
		Glutamate--cysteine ligase (EC 6.3.2.2), divergent, of Alpha- and Beta-proteobacteria type	-	-	-	-	-	-	-	*
	Glutathionylspermidine and Trypanothione	Similarity with glutathionylspermidine synthase (EC 6.3.1.8), group 1	-	-	-	-	-	*	-	-
Periplasmic Stress	Periplasmic Stress Response	HtrA protease/chaperone protein	*	*	-	*	*	*	-	*
		Outer membrane protein H precursor	*	*	*	*	*	*	*	*

* (protein/gene present)
\- (protein/gene absent)

Table S6: Putative prophages predicted by geNomad in the genomes of *Mucilaginibacter* strains isolated from tundra soils. All the prophages which were observed in the strains belonged to the Caudoviricetes class of viruses.

Genome	Topology	Length	Coordinates	Virus score	Hallmarks	Taxonomy
M lappiensis ANJLi2	Provirus	54691	109489-164179	0.9559	6	Caudoviricetes
	Provirus	36280	174233-210512	0.7242	5	Caudoviricetes
Mucilaginibacter tundrae E4BP6	Provirus	20205	4844637-4864841	0.8438	3	Caudoviricetes
	Provirus	23182	2894407-2917588	0.8171	2	Caudoviricetes
	Provirus	6947	-	0.7195	0	Caudoviricetes
Mucilaginibacter saanensis SP1R1	Provirus	52997	403370-456366	0.9652	6	Caudoviricetes
Mucilaginibacter empetricola X4EP1	Provirus	13099	5425787-5438885	0.9007	2	Caudoviricetes

Table S7: Marker genes identified for nitrogen metabolism across Tundra *Mucilaginibacter* strains. The E.C. number of the enzyme, product name and locus of the proteins are mentioned in the table. The genes for ammonia assimilation, assimilatory nitrate and nitrite reduction, and ammonium uptake transport were prominently observed in the strains.

E.C.	Product	*M mallensis* MP1X4	*M frigoritolerans* FT22	*M lappiensis* ANJLi2	*Mucilaginibacter geliditolerans* X5P1	*Mucilaginibacter empetricola* X4EP1	*Mucilaginibacter saanensis* SP1R1	*Mucilaginibacter cryoferens* FT3.2	*Mucilaginibacter tundrae* E4BP6
**Dissimilatory nitrate and nitrite reduction (denitrification)**									
1.7.2.1	Copper-containing nitrite reductase	612309..612749<br>3362260..3362706	(-) (158895..159341)	-	(-) (429824..430273)<br>(-) (2415048..2415488)	3602456..3602872	109935..110393	4382404..4382856	(-) (3161501..3161941)
**Assimilatory nitrate and nitrite reduction**									
1.7.99.4	Assimilatory nitrate reductase large subunit	(-) (4896716..4900225)	-	-	6382943..6386452	4338037..4341546	(-) (3174326..3177862)	(-) (337700..341218)	-
1.7.1.4	Nitrite reductase [NAD(P)H] large subunit	(-) (4904542..4907031)	-	-	6377390..6379879	4332475..4334964	(-) (3182413..3184902)	(-) (76266..78761)	-
1.7.1.4	Nitrite reductase [NAD(P)H] small subunit	(-) (4904150..4904500)	-	-	6379924..6380274	4335009..4335359	(-) (3181944..3182294)	(-) (75827..76147)	-
	Nitrate/nitrite transporter CHU_1319, NarK/U family	(-) (4900249..4901544)	-	-	6381624..6382919	4336719..4338014	(-) (3177868..3179163)	(-) (341422..342711)	-
	ABC transporter, substrate-binding protein (cluster 10, nitrate/sulfonate/bicarbonate)	-	-	-	-	3089400..3090476	-	-	-
**Ammonia assimilation**									
6.3.1.2	Glutamine synthetase type II	(-) (2955991..2957004)	231323..232333	(-) (26328..27338)	927486..928499	(-) (3517452..3518462)	3970521..3971531	(-) (2322882..2323892)	(-) (4053169..4054182)
6.3.1.2	Glutamine synthetase type III, GlnN	(-) (1869466..1871637)	34944..37118	4299..6473	2151860..2154031	4642632..4644806	(-) (3735294..3737468)	5259971..5262145	4200560..4202731

	glutamine synthetase family protein	(-) (2953839..2955197)	232926..234284	(-) (24393..25751)	929306..930664	(-) (3515389..3516786)	3973494..3974852	(-) (2320906..2322264)	(-) (4051149..4052507)
1.4.7.1	Ferredoxin-dependent glutamate synthase	2204919..2206559	-	(-) (173668..175308)	(-) (1708000..1709640)		3710030..3711670	1926741..1928381	3362396..3364036
1.4.1.13	Glutamate synthase [NADPH] small chain	(-) (3308321..3309799)	63900..65378	83415..84893	483720..485198	4673419..4674897	(-) (130808..132286)	3221373..3222851	3139707..3141185
1.4.1.13	Glutamate synthase [NADPH] large chain	(-) (3309802..3314358)	59380..63900	78891..83408	479161..483717	4668899..4673419	(-) (132292..136809)	3216849..3221366	3135176..3139699
	Glutamate synthase		63490..63954		945095..945526	-	-		-
1.4.1.4	NADP-specific glutamate dehydrogenase	2414222..2415658	(-) (103766..105202)	(-) (120247..121683)	(-) (1403067..1404503)	(-) (4716084..4717520)	(-) (5897378..5898814)	(-) (4686516..4687952)	3617752..3619188
1.4.1.2	NAD-specific glutamate dehydrogenase	2414222..2415658	(-) (103766..105202)	(-) (120247..121683)	(-) (1403067..1404503)	(-) (4716084..4717520)	(-) (5897378..5898814)	(-) (4686516..4687952)	3617752..3619188
**Ammonium uptake transport**									
	Ammonium transporter	1872207..1873808<br>(-) (1875894..1877273)<br>(-) (4100455..4101762)	(-) (365404..366711)<br>(-) (32798..34387)	552530..553837<br>(-) (233632..234939)<br>(-) (2081..3703)	2146203..2147582<br>(-) (2149665..2151290)<br>6159877..6161184	(-) (4640485..4642074)<br>(-) (5294500..5295813)	(-) (690249..691556)<br>3738069..3739691<br>(-) (3741971..3743278)	1815453..1816760<br>5253939..5255246<br>(-) (5257713..5259347)	2688224..2689531<br>(-) (4198366..4199988)
	Nitrogen regulatory protein	-	-	-	-	-	(-) (1595531..1599265)	-	-
	Carbon-nitrogen hydrolase	-	-	240066..241592<br>(-) (26052..26993)	(-) (1950627..1951571)	-	991019..992545<br>4120545..4121492	6131438..6132964<br>(-) (103734..104675)	601676..603223
**Proteases**									
	Metallopeptidase	(-)	(-) (316162..318249)	943932..945950	(-) (1440991..1443039)	437455..439509	(+) 636457..638529<br>2730881..2732932	843265..845283	1329656..1331674<br>4242805..4244880

		(1829506..1831569)<br>2375670..2377700<br>(-)<br>(4580361..4582409)	266785..268839<br>268963..269313<br>269301..271016<br>(-)<br>(20032..22050)	304944..306995<br>307207..309258<br>165004..167076<br>45456..47507	2192328..2194391<br>5605562..5607610	(-)<br>(2511903..2513990)<br>4048113..4050125	(-)<br>(3499140..3500963)	(-)<br>(856988..859045)<br>6844275..6846326	
**Amino acid (AA) transport**									
	ABC transporter, substrate-binding protein (cluster 1, maltose/g3p/polyamine/iron)	-	-	-	-	-	-	-	694968..696230
	Na+/H+-dicarboxylate symporter	120711..121946<br>(-)<br>(3503782..3505173)<br>4159539..4160786	159961..161211<br>(-)<br>(278553..279749)<br>188017..189381	8370..9743<br>206189..207409<br>(-)<br>(154355..155659)	238068..239453<br>(-)<br>(6095862..6097103)	2279939..2281186<br>(-)<br>(2398355..2399578)<br>(-)<br>(3574291..3575655)	273584..274804<br>(-)<br>(3139785..3141023)<br>(-)<br>(5944023..5945393)	(-)<br>(2601995..2603185)<br>(-)<br>(4004337..4005731)<br>5621165..5622415	(-)<br>(3888788..3890191)<br>4524110..4525342

Table S8: Cellular fatty acid composition (%) of the novel *Mucilaginibacter* isolates and related species. The related strains are *Mucilaginibacter lappiensis* ANJLI2, *Mucilaginibacter frigoritolerans* FT22 and *Mucilaginibacter mallensis* MP1X4, which were also isolated from tundra sites.

Fatty Acid	E4BP6	FT3.1	SP1R1	X4EP1	X5P1	ANJLI2	FT22	MP1X4
C14:0	0.6	1.1	1	0.9	0.7	0.3	0.4	tr
C15:0	0.5	1.7	3.1	1	0.5	tr	1.2	1.3
C16:0	20.6	23.5	23.3	12.2	17.7	3.7	3.1	4.8
C17:0	0.6	0.6	0.6	1.2	1.8	-	-	-
iso C15:0	30.9	15.6	19.5	32	28.7	18.9	21.9	26
iso-C17:1	16.0	2.9	3.9	7.3	11.9	2.7	4.1	9.5
iso-C15:0 3OH	-	-	-	-	-	2.2	2.9	2.9
iso-C17:0 3OH	5.2	8.8	6.5	4.3	6.3	14.3	16.4	12.5
anteiso-C15:0	0.8	3.3	0.5	0.4	4.7	-	1.6	0.9
C16:1w5c	-	-	-	-	-	6.8	2.7	4
C18:1	1.2	0.9	2.5	1.2	1.6	-	-	-
C16:0 3-OH	3.6	5.3	2.1	2.5	4.7	3.2	0.6	tr
C16:1 ω7c and/or iso-C15:0 2-OH	19.6	35.9	36.1	36.9	21	45	38.7	32.9

Data for strains ANJLI2, FT22, and MP1X4 was from:
Männistö MK, Tiirola M, McConnell J, Häggblom MM. *Mucilaginibacter frigoritolerans* sp. nov., *Mucilaginibacter lappiensis* sp. nov. and *Mucilaginibacter mallensis* sp. nov., isolated from soil and lichen samples. Int J Syst Evol Microbiol 2010; 60:2849-2856.
